# Gestation Dependant Changes in Angiogenic Factors and Their Associations with Fetal Growth Measures in Normotensive Pregnancy

**DOI:** 10.1371/journal.pone.0054153

**Published:** 2013-01-10

**Authors:** Deepali Sundrani, Vinita Khot, Hemlata Pisal, Savita Mehendale, Girija Wagh, Asmita Joshi, Sadhana Joshi

**Affiliations:** 1 Department of Nutritional Medicine, Interactive Research School for Health Affairs, Bharati Vidyapeeth University, Pune, India; 2 Department of Obstetrics and Gynecology, Bharati Medical College and Hospital, Bharati Vidyapeeth University, Pune, India; Université de Montréal, Canada

## Abstract

**Background:**

Earlier studies indicate that altered angiogenesis at birth is associated with poor birth outcome in women with preeclampsia. Now, we hypothesize that the progressive gestation dependant changes in markers of angiogenesis will be more useful to predict birth weight early even in a normotensive pregnancy. This study for the first time examines the association of gestation dependant changes in the levels of maternal angiogenic factors in addition to their levels in cord with birth weight.

**Method:**

Ninety two pregnant women were followed at three different time points: 16–20 weeks, 26–30 weeks and at delivery during pregnancy. Plasma levels of angiogenic and anti angiogenic factors were determined by commercial enzyme-linked immunosorbent assay (ELISA) kits.

**Results:**

Maternal plasma VEGF levels increased (p<0.01) till the second time point and decreased (p<0.05) up to delivery while plasma sFlt-1 levels increased (p<0.01) at delivery. PlGF levels peaked (p<0.01) at second time point and decreased (p<0.01) at delivery. Cord plasma VEGF levels were higher (p<0.01) and sFlt-1 levels were lower (p<0.01) as compared to maternal values at all time points. Maternal plasma VEGF levels at first time point and PlGF levels at delivery were positively (p<0.05 and p<0.01 respectively), while sFlt-1/PlGF ratio at delivery was negatively associated (p<0.05) with birth weight.

**Conclusion:**

Levels of pro- and anti-angiogenic factors may be differentially regulated across gestation. Maternal VEGF levels at early gestation (16–20 weeks) may be predictive of birth weight in healthy term pregnancies.

## Introduction

Vascular growth and remodelling are considered to be central for placental and fetal growth [1]. Angiogenesis is tightly regulated by a number of pro- and anti-angiogenic factors, where the vascular endothelial growth factor (VEGF) family plays a central role. VEGF are a family of structurally related dimeric proteins which play a role in vascular endothelial cell proliferation, survival and migration [2], [3]. Placental growth factor (PlGF) is a major member of the VEGF family that enhances the angiogenic response of VEGF [4], [5]. Biological actions of VEGF and PlGF are primarily mediated through binding to their membrane associated tyrosine-kinase receptors, VEGFR-1 or VEGFR-2 [6]. sVEGFR-1 or soluble fms like tyrosine kinase-1 receptor (sFlt-1) is a soluble receptor and is considered to be an anti-angiogenic factor as it adheres to PlGF and VEGF leading to endothelial dysfunction [2], [7].

Our earlier cross sectional studies in women with preeclampsia at the end of pregnancy indicate altered angiogenesis which was associated with poor birth outcome [8]. However these effects are likely to be confounded by secondary effects of the disease. It has been suggested that the sFlt-1/PlGF ratio could be a biomarker in determining women who are at risk of developing preeclampsia [9]. Therefore, it would be more helpful to examine the levels of these factors in early pregnancy. However, before such studies are undertaken it is imperative to have longitudinal studies which describe changes over time in these angiogenic and anti-angiogenic factors in a normotensive pregnancy.

Few studies which have examined the changes in levels of angiogenic and anti angiogenic factors during normal pregnancy are on a small sample size [10], [11], heterogenous population, varying ethnic and socioeconomic status [12], [13] and smoking population [7], [11], [14], [15], [16]. These factors may lead to altered levels of angiogenic and anti-angiogenic factors and can alter angiogenesis [17], [18].

In fetal life, hypoxia plays an important role in vasculogenesis and angiogenesis [19]. It is known that during the first trimester of pregnancy the human placenta develops in a hypoxic environment which is essential for placental and embryonic development [20]. However, excess hypoxia leads to developmental abnormalities [21], [22]. As gestation increases hypoxia decreases due to increased maternal blood flow in the uterine spiral arterioles. Thus the fetal response to hypoxia plays an important role in fetal development [23]. Since the expression of VEGF is known to be regulated by hypoxia [24] it would be worthwhile examining the association of gestation dependant changes in angiogenic factors in maternal and fetal compartments.

There are however no studies which have examined the association of gestation dependant changes in angiogenic factors or the sFlt-1/PlGF ratio during pregnancy in relation to the levels in cord and their association with fetal growth measures. We hypothesize that markers of angiogenesis in early gestation may be useful to predict birth outcome much earlier even in a normotensive pregnancy. The objective of this study was to determine simultaneously the changes in maternal plasma concentration of these angiogenic (VEGF and PlGF) and anti-angiogenic (sFlt-1) factors over the gestation and their association with cord levels at delivery and fetal growth measures in women with a normotensive pregnancy and delivering at term. All women were extremely well matched for dietary and lifestyle patterns with no smoking, drug or alcohol use to reduce confounders.

## Methods

### Subjects

This longitudinal study was conducted at the Department of Obstetrics and Gynaecology, Bharati Hospital, Pune. This study was approved by the Bharati Vidyapeeth Medical College Institutional Ethical Committee and a written consent was taken from each subject. This study is part of a large ongoing departmental study which recruits all healthy women at 16–20 weeks of gestation. All these women are followed up till delivery and are categorized as preeclampsia if there was presence of proteinuria or high blood pressure and preterm birth if they delivered less than 37 weeks of gestation. However, the current study includes those pregnant women with singleton pregnancy, delivering at term (total gestation ≥37 weeks) and having no medical or obstetrical complications throughout pregnancy. Women were also excluded from the study if there was evidence of other pregnancy complications such as chronic hypertension, type I or type II diabetes mellitus, seizure disorder and renal or liver disease. Thus a total number of ninety two pregnant women were included in this study.

All study participants neither consumed alcohol nor smoked. The women recruited in the study came from a low socioeconomic background and the culture and traditional values of this population do not allow smoking or drinking in women. This has been confirmed when the women were interviewed for demographic and nutritional history. All study participants were from a low socioeconomic group. Kuppuswamy's socioeconomic status scale has been in use as an important measure of socioeconomic status of families in urban communities of India. Kumar et al., 2012 have recently reported Kuppuswamy's socioeconomic scale with an update till the year 2012 with income range [25]. Based on this classification all study participants in this cohort fall in a low socioeconomic class.

In the current study, we recorded dietary intakes using a food frequency questionnaire and has been reported by us earlier [26]. A structured questionnaire was also used to record typical daily routine, which included domestic activities. Based on this we found that all subjects were matched for dietary and lifestyle patterns. All women were routinely given iron tablets as per the National Prophylaxis programme. The gestational age in the current study was determined by last menstrual period (LMP) and ultrasound. The cases with a discrepancy of more than a week between clinical and ultrasound were excluded from the study. Reports by Kalish et al., 2004 suggest that ultrasound is an accurate and useful modality for the assessment of gestational age in the first and second trimester of pregnancy [27].

Data for mothers and blood samples were collected at 3 different time points, 1st time point was at 16–20 weeks, 2nd time point at 26–30 weeks and 3rd time point at delivery. Umbilical cord was also collected just after delivery. Our study design is similar to that reported by other studies which have used similar data points [28], [29], [30]. Among the ninety two pregnant women enrolled at 16–20 weeks of gestation, first time point blood sample was obtained from 92 women, second time point blood sample was obtained from 85 women and at delivery sample was obtained from 85 women since in this population there is precedence for women to migrate to their parent's home for delivery. Umbilical cord blood sample was obtained from 82 women.

### Sample collection and processing

Ten millilitres of maternal venous blood was collected at every time point in EDTA vials. In addition, 10 ml of blood was collected from the umbilical cord just after delivery. The blood was immediately layered on Histopaque (Sigma-Aldrich, St Louis, MO, USA) and centrifuged at 1800 rpm for 35 min to separate the plasma and erythrocytes. The erythrocyte fraction was washed 3 times with normal saline. Then, the plasma and erythrocyte aliquots were stored at −80°C until further analysis.

### Fetal growth measures

Fetal growth measures like birth weight, baby length, baby head circumference and chest circumference were recorded within half an hour after birth. Birth weight was recorded using a digital weighing scale (Zeal medical private limited, India) with an accuracy of 10 g. The length was measured to the nearest 0.1 cm using a portable Infantometer. The head circumference was measured using a measuring fiber glass measuring tape which was placed around the head, just above the eyebrows anteriorly, and around the most prominent bulge posteriorly. The chest circumference was measured using fiber glass measuring tape which was placed around the lower chest.

### VEGF, PlGF and sFlt-1 levels from maternal and cord plasma

Plasma VEGF, PlGF and sFlt-1 levels were measured from both maternal and cord samples using commercial enzyme-linked immunosorbent assay (ELISA) kits (R&D Systems, Minneapolis, MN, USA) and have been described by us earlier [8]. For the sFlt-1 assay, plasma samples were diluted 1∶5 (vol:vol) in calibrator diluent before following the remaining assay procedure of the manufacturer. The detection limit (sensitivity) of the assay was 9 pg/ml for VEGF, 7 pg/ml for PlGF and 13.5 pg/ml for sFlt-1.

Among 92 women from whom blood sample was obtained at the first time point (16–20 week), sufficient plasma was available to analyze VEGF in 77, sFlt-1 in 89 women and PlGF in 63. From second time point (26–30 week), sufficient plasma was available for VEGF measurements in 71 women; sFlt-1 was measured in 76 women and PlGF in 61 women. At the third time point (at delivery), sufficient plasma was available to analyze VEGF in 70, sFlt-1 in 81 and PlGF in 60 women. From umbilical cord blood, sufficient plasma was available to analyze VEGF in 67, sFlt-1 in 74 and PlGF was below the detection limit of the kit.

**Figure 1:**
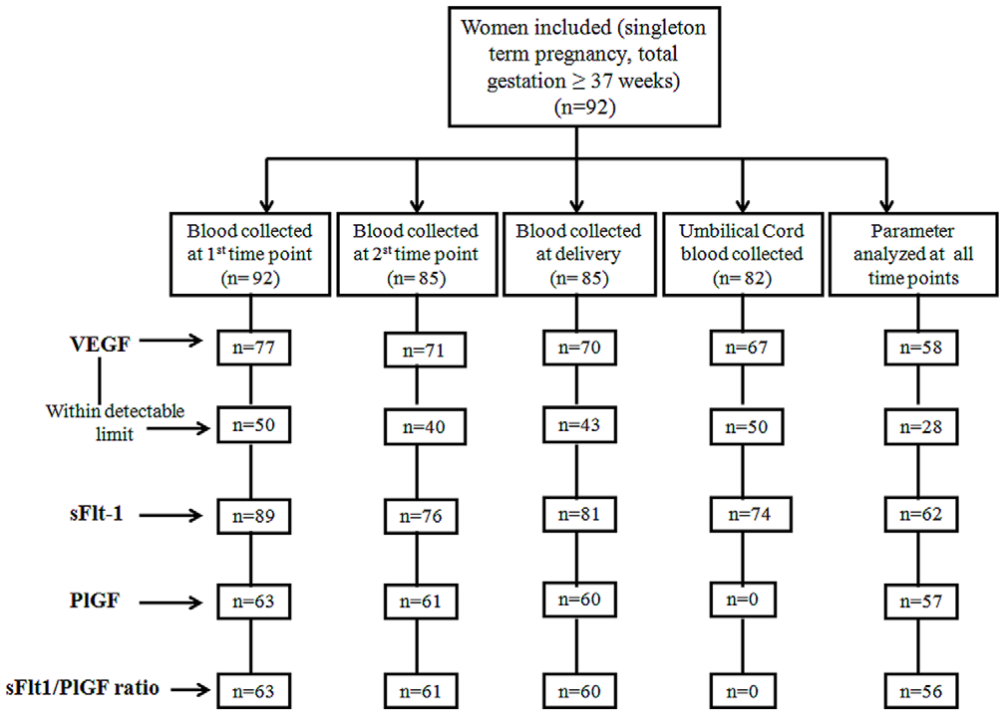
Flow chart for number of samples analyzed at various time points.

At the first time point although VEGF was analyzed from 77 samples, only 50 samples were within the detection limit and 27 samples were below the detection limit. At the second time point from the 71 samples analyzed for VEGF, 40 samples were within the detection limit and 31 were below the detection limit. At delivery from the 70 samples analyzed for VEGF, 43 samples were within the detection limit and 27 were below the detection limit. From 67 cord samples analyzed for VEGF, 50 samples were within the detection limit and 17 samples were below the detection limit (Figure 1). The variable sample number (n) in different measures was a result of insufficient sample volume due to its use for multiple studies ongoing in the lab.

### Statistical analysis

Values are reported as mean ± S.E. The data were analyzed using the SPSS/PC+ package (Version 11.0, Chicago, IL, USA). The data were checked for normal distribution by testing for skewness and kurtosis. Skewed variables were transformed to normality using the following transformations: log to the base 10. Mean values of the various parameters were compared using one way analysis of variance (ANOVA) and the post-hoc least significant difference (LSD) test. For each time point, we assessed the association of circulating angiogenic and anti-angiogenic factors with mother's systolic and diastolic blood pressure, cord levels and with fetal growth measures. Correlation between these variables was studied using Pearson's correlation analysis after adjusting for age, body mass index (BMI) and gestational age at the time of blood sampling.

Statistical analysis was carried out two sets of data. First set of data includes all the women who have participated in the study. Statistical analysis performed on this set of data has been reported in this manuscript. However data was also analyzed in women from whom the parameters were analyzed for all time points as well as in cord. For example VEGF data was analyzed for 28 women from whom VEGF levels were available at all time points. Similarly sFlt-1 data was analyzed for 62, PlGF data for 57 and sFlt-1/PlGF ratio for 56 women. The results of the first set of data have been shown in the figures. However, similar results and trends were observed for the second set of data (data not shown).

## Results

### Demographic characteristics of normotensive mothers and their neonates

Women included in this study had mean age of 25.0±4.0 years, mean body mass index (BMI) of 24.55±3.88 kg/m^2^, mean gestation of 39.3±1.1 weeks, mean systolic BP of 121.1±7.2 mmHg, mean diastolic BP of 78.5±4.5 mmHg and income less than Rs 15,000 per month. The neonates had mean birth weight of 2.9±0.3 kg, length of 47.9±2.6 cm, head circumference of 33.5±1.1 cm and chest circumference of 32.1±1.6 cm.

### Maternal and cord plasma levels of VEGF, sFlt-1, PlGF and sFlt-1/PlGF ratio

#### VEGF

**Figure 2:**
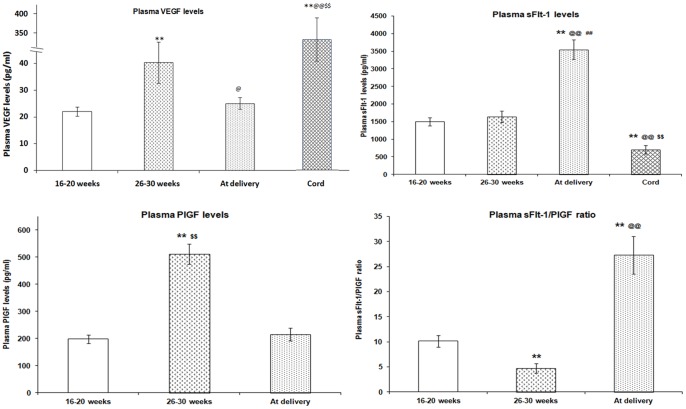
VEGF, sFlt-1, PlGF and PlGF/sFlt-1 levels in maternal plasma at three time points (16–20 week, 26–30 week, at delivery) and in cord plasma. **p<0.01 as compared to T1; ^@^ p<0.05 and ^@@^ p<0.01 as compared to T2; ^$$^ p<0.01 as compared to T3. Type of analysis: ANOVA, posthoc test-LSD.

There was an increase (p<0.01) in maternal plasma VEGF levels from first time point (16–20 weeks) to second time point (26–30 weeks) which decreased (p<0.05) at delivery. In contrast to the maternal plasma levels, the cord plasma VEGF levels were very high (p<0.01) (Figure 2). Similar levels were seen even after including women who were followed for all the time points.

#### PlGF

Maternal plasma PlGF levels followed a bell-shaped curve with advancing gestational age, in which the concentration increased from first time point (16–20 weeks), peaked at second time point (26–30 weeks) (p<0.01) and decreased at delivery (p<0.01) (Figure 2). Similar trend was seen even after including women who were followed for all the time points. In contrast to maternal plasma PlGF levels, cord plasma PlGF levels were below the detection limit.

#### sFlt-1

Maternal plasma sFlt-1 levels were similar at first (16–20 weeks) and second time point (26–30 weeks) while it was significantly higher (p<0.01) at delivery. In contrast to the maternal plasma sFlt-1 levels the cord plasma levels were significantly lower (p<0.01) (Figure 2). Similar trend was seen even after including women who were followed for all the time points.

#### sFlt-1/PlGF ratio

Maternal plasma sFlt-1/PlGF ratio throughout gestation in normal pregnancy followed a curvilinear function in which the ratio was high at first time point (16–20 weeks), decreased at second time point (26–30 weeks) (p<0.01) and then peaked at delivery (p<0.01) (Figure 2). Similar trend was seen even after including women who were followed for all the time points.

### Associations between plasma VEGF and PlGF with blood pressure

There was a positive association between maternal plasma VEGF and PlGF levels with systolic blood pressure (r = 0.342, p = 0.016, n = 49; r = 0.289, p = 0.029, n = 57 respectively) at first time point (16–20 weeks) (Table 1). Similar association was seen even after including women who were followed for all the time points.

**Table 1:** Associations between maternal plasma VEGF, PlGF and sFlt-1 with blood pressure.

	16–20 weeks			26–30 weeks			At delivery		
	n	r	p	n	r	p	n	r	p
***Maternal plasma VEGF (pg/ml)***									
Systolic blood pressure (mmHg)	49	0.274	0.039*	38	0.099	0.553	40	0.030	0.848
Diastolic blood pressure (mmHg)	49	0.004	0.977	38	0.242	0.142	40	0.043	0.793
***Maternal plasma PlGF (pg/ml)***									
Systolic blood pressure (mmHg)	57	0.289	0.029*	57	−0.167	0.210	57	−0.088	0.511
Diastolic blood pressure (mmHg)	57	0.018	0.893	57	0.195	0.141	57	0.203	0.127
***Maternal plasma sFlt-1 (pg/ml)***									
Systolic blood pressure (mmHg)	83	−0.183	0.099	71	0.040	0.742	76	0.172	0.136
Diastolic blood pressure (mmHg)	83	−0.127	0.253	71	0.095	0.431	76	0.046	0.693

Abbreviations: VEGF, vascular endothelial growth factor; PlGF, placental growth factor; sFlt-1, soluble fms-like tyrosine kinase-1.

* p<0.05.

### Associations between maternal plasma VEGF and sFlt-1 with their cord levels

There was a positive association between maternal plasma VEGF levels at second time point (26–30 weeks) and cord plasma VEGF (r = 0.470, p = 0.009, n = 30). There was a positive association between maternal plasma sFlt-1 levels at first time point (16–20 weeks) and at second time point (26–30 weeks) (r = 0.403, p = 0.001, n = 74; r = 0.330, p = 0.007, n = 65 respectively) with cord plasma sFlt-1. In contrast there was a negative association between maternal plasma sFlt-1 levels at delivery and cord plasma sFlt-1 (r = −0.312, p = 0.009, n = 69) (Table 2).

**Table 2:** Associations between maternal plasma VEGF and sFlt-1 with cord plasma levels.

	16–20 weeks			26–30 weeks			At delivery		
	n	r	p	n	r	p	n	r	p
***Maternal plasma VEGF (pg/ml)***									
Cord Plasma VEGF (pg/ml)	36	0.072	0.675	30	0.470	0.009**	33	−0.109	0.546
***Maternal plasma sFlt-1 (pg/ml)***									
Cord Plasma sFlt-1 (pg/ml)	74	0.403	0.001**	65	0.330	0.007**	69	−0.312	0.009**

Abbreviations: VEGF, vascular endothelial growth factor; sFlt-1, soluble fms-like tyrosine kinase-1.

** p<0.01.

### Associations between maternal plasma VEGF, PlGF and sFlt-1/PlGF ratio with fetal growth measures

There was a positive association between maternal plasma VEGF levels at first time point (16–20 weeks) and birth weight (r = 0.361, p = 0.020, n = 39) and levels at second time point (26–30 weeks) with baby length (r = 0.364, p = 0.034, n = 32). There was a positive association between maternal plasma PlGF levels at delivery and birth weight (r = 0.375, p = 0.007, n = 48) and baby length (r = 0.334, p = 0.019, n = 47). There was a negative association between maternal plasma s-Flt/PlGF ratio at delivery and birth weight (r = −0.285, p = 0.045, n = 48) (Table 3). Similar association was seen even after including women who were followed for all the time points.

**Table 3:** Associations between maternal plasma VEGF, PlGF, sFlt-1 and sFlt-1/PlGF ratio with fetal growth measures.

	16–20 weeks			26–30 weeks			At delivery		
	n	r	p	n	r	p	n	r	p
***Maternal plasma VEGF (pg/ml)***									
Baby weight (kg)	39	0.361	0.020*	32	0.053	0.768	37	0.063	0.701
Baby length (cm)	39	0.282	0.074	32	0.364	0.034*	37	0.126	0.446
Head circumference (cm)	39	0.068	0.671	32	0.085	0.631	37	−0.092	0.579
Chest circumference (cm)	39	−0.049	0.763	32	0.015	0.934	37	−0.173	0.291
***Maternal plasma PlGF (pg/ml)***									
Baby weight (kg)	55	−0.069	0.612	53	0.138	0.316	48	0.375	0.007**
Baby length (cm)	55	−0.140	0.299	53	−0.019	0.890	47	0.334	0.019*
Head circumference (cm)	55	−0.035	0.797	53	0.127	0.355	48	0.099	0.496
Chest circumference (cm)	55	0.158	0.239	53	0.085	0.536	48	0.014	0.925
***Maternal plasma sFlt-1 (pg/ml)***									
Baby weight (kg)	77	0.049	0.669	66	−0.038	0.757	64	−0.069	0.584
Baby length (cm)	77	0.040	0.729	66	−0.067	0.585	64	0.005	0.966
Head circumference (cm)	77	−0.048	0.672	66	−0.115	0.351	64	0.083	0.508
Chest circumference (cm)	77	−0.007	0.949	66	−0.081	0.511	64	0.172	0.166
***Maternal plasma sFlt-1/PlGF ratio)***									
Baby weight (kg)	55	−0.010	0.941	52	−0.078	0.575	48	−0.285	0.045*
Baby length (cm)	55	0.032	0.811	52	−0.060	0.667	48	−0.016	0.911
Head circumference (cm)	55	−0.070	0.606	52	−0.191	0.166	48	0.106	0.464
Chest circumference (cm)	55	−0.044	0.744	52	−0.185	0.180	48	0.109	0.450

Abbreviations: VEGF, vascular endothelial growth factor; PlGF, placental growth factor; sFlt-1, soluble fms-like tyrosine kinase-1.

* p<0.05; **p<0.01.

## Discussion

The present study reveals several interesting and important key findings in normotensive women These are: 1) Maternal VEGF and PlGF levels increased from first time point (16–20 weeks) to second time point (26–30 weeks) while there was no change in the sFlt-1 levels. In contrast, maternal VEGF and PlGF levels reduced while sFlt-1 levels increased from second time point (26–30 weeks) to delivery suggesting that levels of pro- and anti-angiogenic factors may be differentially regulated across gestation 2) Maternal VEGF levels at first time point (16–20 weeks) and PlGF levels at delivery were positively associated with birth weight while sFlt-1/PlGF ratio that increased at delivery was negatively associated with birth weight.

There has been increasing interest on the role of circulating pro and anti-angiogenic factors that play an important role in the pathophysiology of various pregnancy complications like preeclampsia, gestational hypertension and spontaneous preterm birth. However, there is need to understand the role of these angiogenic factors across gestation and their association with the levels in the cord as well as birth measures even in a normotensive pregnancy.

In our study there was an increasing trend in maternal plasma VEGF levels from first time point (16–20 weeks) to second time point (26–30 weeks). A possible explanation for this could be that the initial ischemia/hypoxic environment during the 1st trimester trigger VEGF levels to meet the increased demand of mother for placental growth and development. It has been suggested that the increasing trend in VEGF levels across gestation may reflect the development of new blood vessels in the endometrium and trophoblasts in placenta in response to the increased metabolic requirements [31].

Maternal plasma PlGF levels in our study increased till second time point (26–30 weeks) and then declined at delivery. The highest levels at second time point (26–30 weeks) may be a result of a well-established placental circulation. Lower levels of PlGF towards term are suggested to be due to increased sFlt-1 levels as a result of placental ischemia [12], [32]. Similar pattern of PlGF across gestation has been reported earlier [12], [14], [33], [34]. It has been reported that during the initial period of vasculogenesis placental expression of PlGF is moderate [35] and increases after 25 weeks of gestation as angiogenesis switches from branching to non-branching [36].

In our study maternal plasma sFlt-1 levels increase as gestation progresses. This is consistent with studies reported by others that plasma sFlt-1 increases with advancing gestational age [7], [12], [14]. It has been suggested that this may be a result of increasing placental ischemia [12]. Further, it has been proposed that oxidative stress provokes the release of sFlt-1 to a similar or greater extent than hypoxia [37]. Various longitudinal studies have shown increasing oxidative stress during pregnancy [38], [39], [40]. We have earlier reported a positive association between oxidative stress and sFlt-1 in preeclampsia [8]. Further, maternal plasma sFlt-1/PlGF ratio followed a curvilinear function in which the ratio decreased from first time point (16–20 weeks) to second time point (26–30 weeks) and then peaked at delivery. This is consistent with other reported studies [7], [12].

Furthermore, in our study the cord plasma VEGF levels were very high as compared to the maternal levels. This suggests a differential functional role of VEGF in maternal and fetal compartments. We have earlier reported that the levels of angiogenic factors at delivery are differently regulated in the maternal and cord samples in preeclampsia [8]. Cord sFlt-1 levels were lower as compared to maternal levels at every time point during gestation. It has been reported that there is a polarized secretion of sFlt-1 into the maternal but not in the fetal compartment for angiogenesis [41].

**Figure 3:**
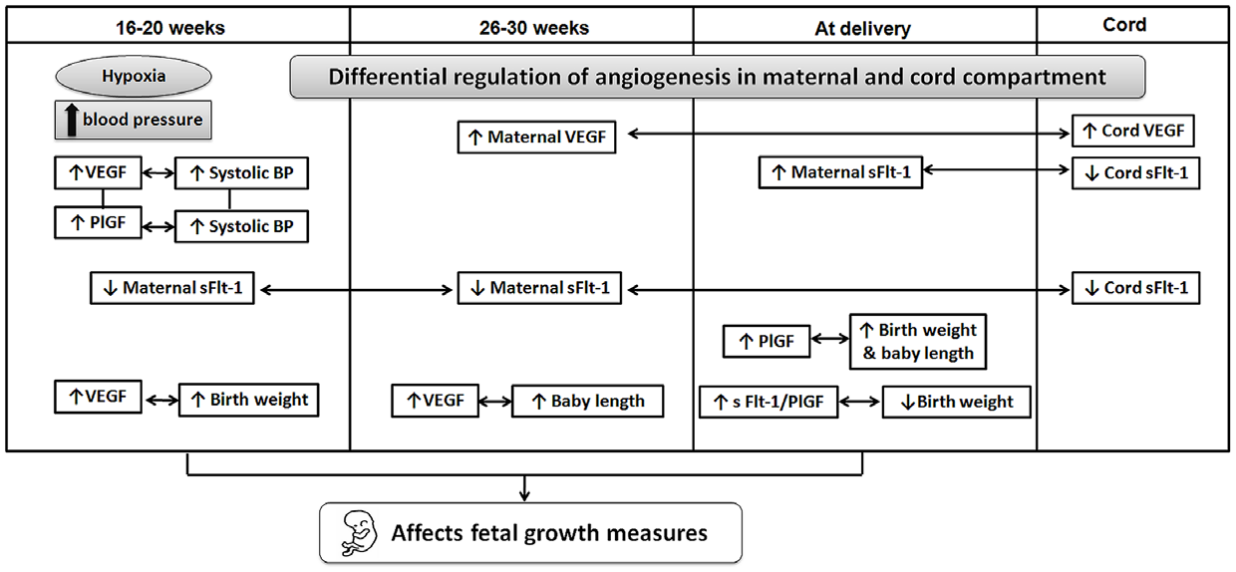
Schematic diagram for the association of maternal angiogenic factors with cord angiogenic factors, blood pressure and fetal growth measures.

In our study, there was a positive association between maternal plasma VEGF and PlGF levels only at first time point (16–20 weeks) with systolic blood pressure (Figure 3). During early pregnancy, it is known that the placenta develops in a hypoxic environment. Hypoxia has been shown to increase blood pressure in animals [42]. Systolic blood pressure has been reported to increase in pregnant mice treated with hypoxia inducible factor alpha [43]. Further, maternal plasma sFlt-1 levels were positively associated at first time point (16–20 weeks) and second time point (26–30 weeks) but were negatively associated at delivery with the cord sFlt-1 levels.

Lastly, in our study, maternal plasma VEGF and PlGF levels at first time point (16–20 weeks) and at delivery were positively associated with birth weight (Figure 3). Early placentation defects, including impaired maternal spiral artery remodelling and placental vascularisation have been demonstrated in pregnancy complications like preeclampsia and preterm births [44], [45], [46], [47]. Our findings suggest that early maternal VEGF levels determine appropriate placentation which ensures optimal fetal growth. Further, our data indicates that maternal sFlt-1/PlGF ratio (an indicatior of vascular dysregulation) at delivery was negatively associated with birth weight. This supports our earlier findings of a negative association of maternal sFlt-1 level with baby weight, head and chest circumference in the preeclampsia [8]. The positive association of maternal VEGF levels at first time point with birth weight supports our hypothesis that markers of angiogenesis may be useful to predict birth weight early in pregnancy (16–20 weeks) even in a normotensive pregnancy. One limitation of the study is that the sample size is relatively small and we could not rule out the possibility of false positive or negative findings in multiple comparisons.

To summarize, our findings indicate that levels of angiogenic factors are differentially regulated throughout gestation depending upon the differential physiological needs of the mother and fetus. Dysregulation in angiogenesis even in normotensive pregnancy may affect presently unknown and/or marginally consequential birth outcome. However, the patterns of these angiogenic factors could possibly be used as a biomarker in identifying pregnancy complications and predicting the fetal outcome.
